# Community engagement to enhance trust between Gypsy/Travellers, and maternity, early years’ and child dental health services: protocol for a multi-method exploratory study

**DOI:** 10.1186/s12939-016-0475-9

**Published:** 2016-11-14

**Authors:** Alison McFadden, Karl Atkin, Kerry Bell, Nicola Innes, Cath Jackson, Helen Jones, Steve MacGillivray, Lindsay Siebelt

**Affiliations:** 1 School of Nursing and Health Sciences, University of Dundee, 11 Airlie Place, Dundee, DD1 4HJ UK; 2 Department of Health Sciences, University of York, Heslington, York, YO10 5DD UK; 3 Dental Hospital and School, University of Dundee, Park Place, Dundee, DD1 4HR UK; 4 Leeds Gypsy and Traveller Exchange, Crown Point House, 167-169 Cross Green Lane, Leeds, LS9 0BD UK

**Keywords:** Gypsy/Travellers, Roma, Trust, Community engagement, Maternity services, Early years’ services, Child dental health services, Case study, Multi-method research, Socially-excluded populations

## Abstract

**Background:**

Gypsy/Travellers have poor health and experience discrimination alongside structural and cultural barriers when accessing health services and consequently may mistrust those services. Our study aims to investigate which approaches to community engagement are most likely to be effective at enhancing trust between Gypsy/Travellers and mainstream health services.

**Methods:**

This multi-method 30-month study, commenced in June 2015, and comprises four stages.

1. Three related reviews: a) systematic review of Gypsy/Travellers’ access to health services; b) systematic review of reviews of how trust has been conceptualised within healthcare; c) realist synthesis of community engagement approaches to enhance trust and increase Gypsy/Travellers’ participation in health services. The reviews will consider any economic literature;
2. Online consultation with health and social care practitioners, and civil society organisations on existing engagement activities, including perceptions of barriers and good practice;
3. Four in-depth case studies of different Gypsy/Traveller communities, focusing on maternity, early years and child dental health services. The case studies include the views of 32–48 mothers of pre-school children, 32–40 healthcare providers and 8–12 informants from third sector organisations.
4. Two stakeholder workshops exploring whether policy options are realistic, sustainable and replicable.

Case study data will be analysed thematically informed by the evaluative framework derived from the realist synthesis in stage one.

The main outputs will be: a) an evaluative framework of Gypsy/Travellers’ engagement with health services; b) recommendations for policy and practice; c) evidence on which to base future implementation strategies including estimation of costs.

**Discussion:**

Our novel multi-method study seeks to provide recommendations for policy and practice that have potential to improve uptake and delivery of health services, and to reduce lifetime health inequalities for Gypsy/Travellers. The findings may have wider resonance for other marginalised populations. Strengths and limitations of the study are discussed.

**Trial registration:**

Prospero registration for literature reviews: CRD42015021955 and CRD42015021950

UKCRN reference: 20036

## Background

In 2008, the World Health Organisation Commission on Social Determinants of Health [1] called for ‘closing of the gap’ in health inequalities within a generation. Reducing health inequalities has been a priority for successive UK governments [2]. The needs of the most marginalised groups have however, been neglected. Gypsies and Travellers are one socially excluded group where evidence for improving health is weakest [3]. It is estimated that there are 150,000–300,000 Gypsy/Travellers in the UK [4], this however is likely to be an underestimate. Due to widespread stigma and discrimination, many Gypsy/Travellers do not disclose their identity [5]. This paper provides an overview of a multi- component study that aims to strengthen the evidence regarding how to improve uptake and delivery of health services and thereby reduce health inequalities for Gypsy/Travellers.

We use the term ‘Gypsy/Travellers’ to include all those with a cultural tradition of, and commitment to nomadism, including those who live permanently or temporarily in settled housing. This broad definition includes individuals from different socio-cultural backgrounds including Romany (English) Gypsies, Irish Travellers, Scottish Gypsy/Travellers and Eastern European Roma communities. However, there are contested definitions of Gypsy/Travellers reflecting complex cultural and/or linguistic differences between communities [6]. Therefore there are likely to be different health needs and experiences of health care between and within diverse Gypsy/Traveller communities [7].

Although Gypsy/Traveller communities are diverse, and robust evidence of health needs is lacking due to unknown population size and lack of systematic monitoring [8, 9], there is consensus that Gypsy/Travellers in the UK have poorer health and lower life expectancy than the general population and other disadvantaged groups [7, 8, 10–15]. This includes increased maternal and child mortality [8, 13, 16], and in children, high rates of accidental injury, infections and accident and emergency department attendance [11, 17]. Studies have found low uptake of preventative health services including childhood immunisations [18–21], significantly increasing risk of preventable disease [22, 23]. Gypsy/Travellers have poor dental health with high unmet need for dental care [24, 25].

Some of the reasons why Gypsy/Travellers are vulnerable to poor health outcomes, even when compared to other disadvantaged groups include poor living conditions, high rates of homelessness, low educational achievement, social exclusion and widespread prejudice and discrimination [26]. Gypsy/Travellers also face many barriers to accessing healthcare. For some, a mobile lifestyle is key [16], however, poor access is also experienced by settled Gypsy/Travellers. This is underpinned by complex factors including stigmatisation and lack of understanding by healthcare staff [10, 12, 27]. Reported cultural barriers include normalisation of ill-health and pride in self-reliance [28]. However, it is unclear how these interact with social exclusion and poverty [29].

These multiple factors alongside poor quality care that does not meet healthcare needs may lead to low expectations and mistrust of health services and healthcare personnel [27, 30]. Trust in services and personnel is associated with increased utilisation of healthcare, and improved health behaviours and quality of care [31–33]. Community engagement strategies have the potential to enhance trust and ensure services are tailored to the needs of specific populations [34–36]. “Community engagement” is one of several overlapping terms (others include “community involvement”, “community participation”, and “community development”) used to describe activities that are aimed at enabling communities to participate in decisions that affect their lives and improve their health and wellbeing, including planning, design, delivery and evaluation of health services [34–36].

### Aims and objectives

Our research investigates which approaches to community engagement are likely to enhance trust between Gypsy/Travellers and mainstream health services. The focus is maternity services, early years’ health services and child dental health services. The objectives are to:

1. describe activities and methods used to engage Gypsy/Travellers in health services and to assess the extent to which they focus on developing trust;
2. investigate the extent to which different engagement activities used by health services enhance trust and increase uptake of maternity services, early years’ services and child dental health services by Gypsy/Travellers;
3. examine the knowledge, attitudes/beliefs and experiences of Gypsy/Travellers of maternity services, early years’ services and child dental health services;
4. identify different approaches to enhancing Gypsy/Travellers’ trust in maternity services, early years’ services and child dental health services and explore the implications for policy and practice;
5. estimate the potential implementation costs of different approaches to enhancing Gypsy/Travellers’ trust in maternity services, early years’ services and child dental health services; and
6. explore whether community engagement approaches that work to enhance Gypsy/Travellers’ trust in maternity services, early years’ services and child dental health services are potentially applicable to other health services/vulnerable communities.

## Methods

### Study design and overview

**Fig. 1:**
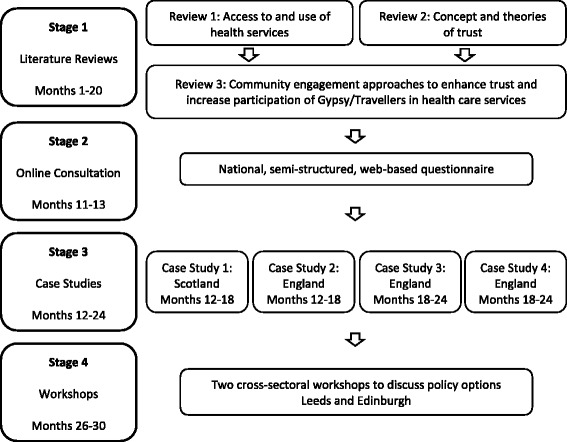
Study flow chart

This multi-method 30-month study (June 2015 to November 2017) comprises four interlinked stages. See Fig. 1 for an overview.

The study team are being advised by two advisory groups; a Stakeholder Advisory Group comprising health professionals, policy advisors and academics, and a User Advisory Group, hosted by Leeds Gypsy and Traveller Exchange (Leeds GATE), comprising women representing Romany Gypsy, Irish Traveller and Eastern European Roma communities.

### Stage one: literature reviews

Review one is a systematic review of all available primary empirical literature on how, why and where Gypsy/Travellers seek help from and engage with healthcare services.

#### Search

In May 2015, we searched 21 online databases: MEDLINE (via OVID), Embase (via OVID), CINAHL (via EBSCO), Cochrane Database of Systematic Review, Database of Abstracts of Reviews of Effects, Health Technology Assessment database, CENTRAL, Social Science Citation Index (via Web of Knowledge), PsycINFO (via OVID), HMIC (via OVID), ASSIA (via Proquest), Social Policy and Practice (via OVID), Bibliomap (via the EPPI-Centre databases), DoPHER (via the EPPI-Centre databases), TRoPHI (via the EPPI-Centre databases), the Campbell Library, Social Care Online and the British Nursing Index (via Proquest), Research Councils UK – Gateway to Research, OAIster and OpenGrey. In addition, to identify work-in-progress and unpublished studies, a focused Google search was conducted. Reference lists of relevant literature reviews were examined to locate further studies. Search terms, developed with an Information Specialist, combined thesaurus and free-text terms. The search structure was (Gypsy/Traveller communities) AND (general healthcare services OR maternal and child healthcare services OR child dental health care services OR community engagement interventions).

#### Eligibility criteria

Publications were included if they reported methods and findings of a primary study, focused on Gypsy/Travellers, included data that illuminated how, why and where Gypsy/Travellers engage with health care services and were published in English after the year 2000. All study designs were included.

#### Selection of studies

Title and abstracts were screened independently by two reviewers and discrepancies discussed with a third reviewer. Full texts of publications appearing to meet the inclusion criteria were assessed independently by two reviewers and discrepancies discussed with a third reviewer.

#### Data extraction and synthesis

For each study meeting the eligibility criteria, data were extracted by one reviewer and checked by a second reviewer regarding methods, aims and specific findings related to the review question. A detailed narrative of the findings will be reported.

#### Output

An evidence matrix indicating key findings and the robustness of methodology, accompanied by a narrative synthesis is the key output of this review. Review one also provides a sampling frame to feed relevant studies into the realist synthesis of community engagement approaches [37]. Finally, we also conducted an appraisal of the economics literature applying focus to any economic evaluations or discussions of cost associated with engagement programmes. Economics literature was primarily identified via the first search though an additional search was also undertaken using NHS EED, the only remaining database for economic evaluations (published until 2014). Review one is in the write-up phase.

Review two is a systematic review of secondary (review) literature to examine how ‘trust’ has been conceptualised and theorised in any healthcare setting. Trust is a complex term, frequently used but rarely defined. We are particularly interested in describing frameworks/models that may be relevant in explaining the relationship between vulnerable communities and mainstream health and social care services. Trust is however, a challenging term to search for (a recently updated Cochrane review on interventions to enhance trust retrieved 14057 records for initial screening [38]). Since we were interested in understanding and describing the concept of trust within a health care context generally, we focused on secondary literature.

#### Search

We searched 15 online databases in May 2015: MEDLINE (via OVID), Embase (via OVID), CINAHL (via EBSCO), Cochrane Database of Systematic Review, Database of Abstracts of Reviews of Effects, Health Technology Assessment database, Social Science Citation Index (via Web of Knowledge), PsycINFO (via OVID), HMIC (via OVID), ASSIA (via Proquest), Social Policy and Practice (via OVID), Bibliomap (via the EPPI-Centre databases), DoPHER (via the EPPI-Centre databases), TRoPHI (via the EPPI-Centre databases), the Campbell Library. The search structure was: “trust” synonyms AND “systematic review” synonyms.

#### Eligibility criteria

Systematic and non-systematic reviews were included if their primary focus was describing or exploring the concept of trust within a health care context, and were published in English after the year 2000

#### Study selection

Title and abstracts were screened independently by two reviewers and discrepancies discussed with a third reviewer. Full texts of publications appearing to meet the inclusion criteria were assessed independently by two reviewers and discrepancies discussed with a third reviewer.

#### Data extraction and synthesis

Data were extracted for each eligible study by one reviewer and checked by a second reviewer regarding: methods; review aims; and key findings specifically related to understanding, describing or exploring trust. A detailed narrative synthesis of the findings is currently under construction.

Review three is a realist synthesis of community engagement approaches to enhance trust and increase Gypsy/Travellers’ participation in health services. Four hypotheses, derived from published literature, were developed to provide initial direction for the review:

1. Community engagement is an effective and cost-effective strategy for enhancing the confidence and trust of Gypsy/Travellers in mainstream services [34];
2. Approaches to community engagement that work to enhance trust and increase uptake of services with some participants may not work with Gypsy/Travellers because of the longstanding experience of social exclusion and discrimination, low education and literacy levels and mistrust of authority [39];
3. Successful community engagement will be underpinned by genuine involvement of community members (i.e. not tokenistic), honest appraisal of what can be achieved (not raising expectations that cannot be met) and continuity of trusted personnel [40].
4. Community engagement between Gypsy/Travellers and mainstream health services can be facilitated effectively by civil society Gypsy/Traveller organisations [8, 39].

Realist synthesis is appropriate for understanding complex interventions, in this case the interaction between trust and community engagement. Realist reviews focus on developing theories of what works for whom and in what circumstances thereby accounting for context, mechanisms and outcomes in the process of systematically synthesising relevant literature [41]. Our realist synthesis will draw on data derived from reviews one and two, but will also include purposive additional searching [37] for literature that focuses on engagement approaches with Gypsies, Travellers and Roma. The output of the realist synthesis will be an evaluative framework for explaining and understanding the complex and multi-faceted nature of engagement with health services. We plan to involve the study Stakeholder Advisory group in further stages of the review process. Review three is underway.

### Stage two: online consultation

A semi-structured, web-based questionnaire will be purpose designed to elicit views on how to enhance trust in mainstream services; the range of activities/methods used by maternity, early years’ and child dental health services to engage Gypsy/Travellers and any associated costs; perceptions of the success of different approaches to developing trust; and barriers to, and suggested strategies for, enhancing trust, including examples of good practice. The questions will be based on the aims of the study, findings of the literature reviews, and the views of the Stakeholder Advisory Group. The consultation will be delivered using the Bristol Online Survey tool [42], and will be disseminated by e-mail. We aim to include the views of three main groups, from across the UK, through purposive sampling:

1. Individuals working in civil society organisations who represent or advocate for Gypsy/Traveller communities. These include UK-wide organisations such as Friends, Family and Travellers; National Federation of Gypsy Liaison Groups; and local/regional groups such as Traveller Movement (London); Derbyshire Gypsy Liaison Groups; One voice for Travellers (Cambridgeshire); Roma Support Group (London); Romani Arts Company (Wales) An munia Tober (Northern Ireland) and Article 12 Young Gypsy Lives (Scotland). We will also include organisations who represent/advocate for users of maternity users (nct – formerly known as the National Childbirth Trust), and children (Save the Children UK; Children’s Society). We were unable to identify any civil society organisations focusing on child dental health.
2. Health and social care practitioners delivering maternity, early years’ and child dental health services (e.g. midwives, health visitors, general practitioners, and community dentists, who work with Gypsy/Travellers communities). We aim to include healthcare practitioners who have a specialist role regarding service provision for Gypsy/Travellers, and those who provide care for Gypsy/Travellers as part of mainstream services. We will reach these practitioners through professional organisations and networks such as Midwifery Supervisors network; Infant Feeding Leads network; Health Visitors Institute; Royal College of General Practitioners; Royal College of Paediatrics and Child Health; Faculty of Public Health; British Dental Association and British Society of Paediatric Dentistry.
3. Local policymakers and health and social care service commissioners (e.g. Directors of Public Health and Dental Public Health, health improvement specialists, health inequality teams, clinical commissioning groups and Local Authorities).

Analysis of the online consultation will include: proportions of respondents who agree/disagree with evidence–derived statements; and thematic analysis of free text questions including exploration of similarities and differences between different stakeholders.

The online consultation findings will: a) inform the selection of case studies, i.e. if a successful approach to community engagement with Gypsy/Travellers is identified, we may select the location as a case study site; b) provide a national context to locate the findings of the case studies; and c) provide a community of interest for dissemination of the study findings.

### Stage three: case studies

#### Settings and participants

This stage comprises multiple case study design to explore in-depth community engagement and trust in healthcare for Gypsy/Travellers [43]. The unit of analysis is the approach to engagement between health services and Gypsy/Traveller communities within a locality. Each case study involves interviews, focus group discussions and documentary analysis. Four case studies will be selected purposively to reflect the diversity of Gypsy/Travellers communities, different approaches to community engagement, and examples of good practice regarding maternity, early years’ or child dental health services (identified through the realist synthesis and online consultation). Three case studies will be in England and one in Scotland to reflect the larger population of Gypsy/Travellers in England and to meet the funders’ remit of advising policymakers in England. The selection of a case study in Scotland strengthens the methodology because there are differences between healthcare structures and remuneration in England and Scotland that could be significant.

Overall, the case studies will include English/Romany Gypsies, Irish Travellers, Scottish Gypsy/Travellers and Eastern European Roma migrants. They will be conducted in two phases of six months. Lessons learned from the first two case studies, for example approaches to recruitment or revisions to interview topic guides, will inform the conduct of the second two case studies.

Our purposive sample strategy is designed to reflect the diversity of Gypsy/Traveller populations living in the UK. We aim to recruit mothers who live in permanent housing, and in authorised and unauthorised sites, and those following a nomadic lifestyle. Where the mother wishes, we will include other family members in interviews Health practitioners will be recruited purposively to include those working in maternity, early years’ and child dental health services. Finally we will include key informants from civil society organisations that are involved in community engagement activities with Gypsy/Travellers. See Table 1 for an overview of the proposed numbers of participants and data generation methods.

**Table 1:** Target numbers of participants in case studies

Participants	Data generation method	Each case study	Total across four case studies
Mothers of pre-school children	Face-to face interviews	8–12	32–48
Health and social care practitioners	Focus group discussion	6–8	24–32
	Telephone interviews	2–4	8–16
Key informants from civil society organisations	Telephone interviews	2–4	8–16

The case studies will include analysis of documents, sourced through NHS and civil society organisations, websites, social media and from the research participants, related to methods and activities used by health services and civil society organisations to engage Gypsy/Travellers.

#### Access and recruitment

There are challenges in recruiting participants from marginalised communities. In each case study we will identify relevant civil society organisations, community workers, local authority or NHS frontline health and social care workers as gatekeepers who can identify potential participants. Leeds GATE will facilitate recruitment through their networks. We are developing relationships both for circulating the online consultation (stage two) and facilitating recruitment to case studies. We will liaise with individuals and organisations working with Gypsy/Travellers with whom we have established links and who are familiar with research process through a previous study [44]. The Stakeholder Advisory Group will identify additional organisations and specialist services to enahnce recruitment. The gatekeepers will facilitate recruitment of health and social care practitioners and key informants from civil society organisations.

#### Generating research material

##### Mothers of pre-school children

We will conduct semi-structured, face-to-face interviews. The interview topic-guide will focus on perceptions of trust, views, experiences and awareness of maternity, early years’ and child dental health services including barriers to service use, experiences of community engagement activities, and suggestions for ways of improving services.

##### Health and social care practitioners

We will conduct focus group discussions with telephone interviews as a contingency for those unable to attend a focus group [45]. The topic guide will include participants’ experiences of service provision for Gypsy/Traveller communities, barriers to providing quality services, organisational context, examples of good practice in terms of engagement and developing trust with Gypsy/Traveller communities and cost implications.

##### Key informants from civil society organisations

We will conduct telephone interviews, focusing on views and experiences of different approaches to community engagement, barriers and suggested strategies for increasing trust between Gypsy/Travelers and mainstream health services.

The locations of interviews and focus group discussions will be negotiated with participants. All interviews and focus groups will be audio-recorded with the participants’ written consent and transcribed for analysis. Where necessary, interviews with participants from Eastern European Roma backgrounds will be undertaken by a bilingual researcher who will transcribe and translate the audio-recording.

### Analysis of research material

We will analyse data thematically, informed by the evaluative framework derived from the realist synthesis in stage one. The research material from each case study will be analysed and reported independently before comparing similarities and differences across case studies [46]. We will analyse diverse participant experience to avoid essentialist interpretations based on particular cultural groups [47]. NVivo 10 Software [46] will be used to manage the data.

Costs incurred by health and social care services will be estimated for each approach identified and represented as per family/per individual depending on the nature of the cost. All potential sources of costs will be identified, for instance cost of a visit from an appropriately trained practitioner. Cost data will be drawn from systematic review evidence and standard costing sources [47]. Although the results will only provide a conservative estimate of the costs associated with each approach, such knowledge is important to guide decision-making and future trials. If data is sufficiently rich, a theoretical cost-benefit analysis could be included using real life experiences of Gypsy/Travellers to estimate the potential benefits through cost savings.

The findings of the three completed stages of the research (reviews, online consultation and case studies) will be synthesised, using a triangulation protocol [48], to draw up a list of approaches to community engagement for enhancing Gypsy/Travellers’ trust in mainstream services. This will be done at the data interpretation phase [49]. A ‘convergence coding matrix’ will be created to display the different sets of findings informed by the evaluative framework developed from the realist synthesis.

### Stage four cross-sectoral workshops

Two cross-sectoral workshops will present the draft policy options/recommendations to diverse stakeholders. This approach ensures that options/recommendations culminating from research reflect the realities and constraints of policy and practice [50]. Furthermore, the workshops will create a community of interest for dissemination. Up to 40 stakeholders will be invited to attend (or nominate a deputy) including: representatives from civil society organisations; frontline maternity and early years’ health services and children’s dental health services staff, service managers and commissioners, national and local policymakers, representatives from Local Authorities, and members of the User and Stakeholder Advisory Groups. Detailed field notes along with materials from the groupwork and plenary sessions, will be synthesised and included in the final report.

Workshop participants will consider:

- the importance, acceptability, feasibility, replicability and sustainability of recommendations;
- barriers to and positive strategies for implementation of recommendations;
- possible consequences and costs of different policy options;
- how policy and practice options might work in different healthcare settings (e.g. mental health, adult dental services) and for other vulnerable populations (e.g. vulnerable migrants, homeless).

### Public and patient involvement

It would not be possible to undertake this study without the involvement of Gypsy/Travellers. The study team includes the Chief Executive Officer of Leeds GATE, who is hosting the User Advisory Group whose involvement will include: input to the evaluative framework derived from the realist synthesis; development of participant information sheets and consent forms; advice on recruitment, topic guides for interviews and focus group discussions; interpretation of findings, and dissemination activities. In each case study location, we will identify two members of the local Gypsy/Traveller community to advise on the conduct of the research and any local issues of relevance, for example access, recruitment, and locally-tailored participant information sheets. We will support members of the User Advisory Group and local case study community members through two advocacy-training events in the first and second years of the project. The participatory events will bring together community members, researchers and members of civil society organisations.

### Dissemination

The main output will be a report detailing: a) an evaluative framework of Gypsy/Travellers’ engagement with health services; b) recommendations for policy and practice on how to enhance trust and improve the acceptability of health services to Gypsy/Travellers; and c) evidence on which to base future implementation strategies including estimation of costs of policy options. To increase impact, we will disseminate widely through written summaries, social media, and academic and professional conferences and publications. This will include: to Gypsy/Travellers communities led by the User Advisory Group; to research participants, and more widely through the network of civil society organisations developed from the online consultation and stakeholder workshops. Short articles will be written for magazines/newsletters. More detailed summaries will be prepared for health and social care organisations and disseminated to relevant professional organisations.

## Discussion

This multi-component study seeks to explore ways of improving the uptake and delivery of health services and thereby reducing health inequalities for Gypsy/Travellers who are marginalised in the UK and across Europe [51]. The multi-method approach will combine data from a variety of perspectives including Gypsy/Travellers, health professionals and civil society organisations to provide policy recommendations to enhance trust and improve the acceptability of health services to Gypsy/Travellers

Although we have chosen to focus on maternity, early years’ and child dental health services as exemplars of mainstream health services, the findings may have resonance for other health services. Issues of trust and engagement are likely to be determinants of differential uptake of health services for other marginalised populations such as homeless people and refugees/asylum seekers. Thus, our findings may have broader application. The robust methods of public and patient involvement will help to ensure that the research is conducted ethically. The involvement of stakeholders, particularly through the workshops will increase the likelihood that final recommendations reflect the realities and constraints of policy and practice. Through the online consultation and our approach to selecting the case studies we aim to provide best practice guidance.

Our study has several challenges and limitations. Trust and engagement are terms with multiple meanings. We planned the detailed literature reviews to develop theoretical understanding of these concepts which can then be explored in case studies and workshops. We anticipate that the explanatory framework will address different meanings of trust and engagement especially when these might differ between Gypsy/Traveller populations and health services. Within the constraints of the time and funding we are limited to four case studies which will be selected on the basis of good practice. This may reduce our ability to reflect on lessons-learned from approaches that have not worked. By the very nature of the marginalisation and discrimination experienced by Gypsy/Travellers in UK society, it is likely that recruitment to our study will be challenging and require multiple approaches. The ability of the researchers to develop trusting relationships first with gatekeepers and then with participants will be critical to the quality of the findings. We may not be able to recruit those who are most vulnerable e.g. those who do not engage with civil society organisations and/or those living in unauthorised encampments. Health professionals who participate are likely to be those who have an interest in this population group and therefore may not represent all mainstream practitioners. Despite these caveats, our study will add to the evidence-base of what works to increase trust and engagement between marginalised populations and mainstream health services.
